# Digital Pathology with AI for Cervical Biopsies: Diagnostic Accuracy at the CIN2+ Threshold

**DOI:** 10.3390/cancers17233808

**Published:** 2025-11-27

**Authors:** Anja Kristin Andreassen, Elin Mortensen, Roy Stenbro, Øistein Sørensen, Sveinung Wergeland Sørbye

**Affiliations:** 1 Department of Clinical Medicine, Faculty of Health Sciences, UiT The Arctic University of Norway, 9019 Tromsø, Norway; aan159@post.uit.no; 2 Department of Medical Biology, Faculty of Health Sciences, UiT The Arctic University of Norway, 9019 Tromsø, Norway; elin.mortensen@unn.no; 3 Department of Clinical Pathology, University Hospital of North Norway, 9019 Tromsø, Norway; 4 EagleEye, 0655 Oslo, Norwaysorenso@gmail.com (Ø.S.)

**Keywords:** digital pathology, artificial intelligence, deep learning, whole-slide imaging, cervical intraepithelial neoplasia, adenocarcinoma in situ, diagnostic accuracy, observer agreement, p16 immunohistochemistry

## Abstract

**Background/Objectives**: Histopathologic grading of cervical biopsies is subject to interobserver variability, particularly at the CIN2+ treatment threshold. We evaluated a deep learning system (EagleEye) for detecting CIN2+ (CIN2, CIN3, ACIS, invasive carcinoma) on hematoxylin–eosin (H&E) whole-slide images (WSIs) and compared its performance with independent pathologists, including an AI-assisted workflow. Spatial correspondence with p16 staining was preliminarily assessed. **Methods**: Ninety-nine archived cervical punch biopsies from a single university hospital, originally diagnosed as Normal (n = 19), CIN1 (n = 20), CIN2 (n = 20), CIN3 (n = 20), or adenocarcinoma in situ (ACIS; n = 20), were digitized in a deliberately spectrum-balanced design. The original sign-out (P1), a second gynecologic pathologist (P2, microscope and digital), EagleEye alone (EE), and an AI-assisted read (EE + P2) served as diagnostic conditions. Outcomes were dichotomized as <CIN2 vs. CIN2+. Agreement was evaluated using Cohen’s κ and sensitivity/specificity (95% CI) under pre-specified internal reference standards. In 30 cases with prior p16 staining, visual correspondence between AI heatmaps/tiles and p16-positive epithelial domains was recorded. **Results**: Agreement between P1 and EagleEye was moderate (κ = 0.67), while P2 showed high internal consistency (κ = 0.86) and good agreement with P1 (κ = 0.78). Using P1 as reference, EagleEye detected CIN2+ with 93.3% sensitivity and 71.8% specificity. When the AI-assisted consensus (EE + P2) was used as an augmented internal comparator, P1 showed 83.8% sensitivity and 100% specificity, indicating that the human-in-the-loop workflow identified additional CIN2+ cases that had been signed out as <CIN2 by P1, particularly near the CIN1/CIN2 boundary. In a subset of cases originally classified as CIN3 by P1, EagleEye flagged squamous cell carcinoma (SCC); several of these were confirmed as SCC by expert review (EE + P2). In ACIS, EagleEye under-called relative to pathologists but improved after adjudication. p16-to-AI spatial correspondence ≥70% was observed in 73.3% of evaluated cases. **Conclusions**: In this single-centre, spectrum-balanced cohort, EagleEye achieved high CIN2+ sensitivity and substantial agreement with expert readers in a human-in-the-loop workflow. The main added value was internal case-finding of treatment-relevant lesions when AI assistance was available, while final diagnoses remained with the pathologist.

## Simple Summary

Diagnosing precancerous cervical lesions on biopsies can be challenging, and pathologists may disagree on borderline cases. In this study, we evaluated an artificial intelligence (AI) system that analyzes digital whole-slide images and compared it with routine pathologist readings, as well as with the same expert pathologist using AI as decision support. In this spectrum-enriched series, the AI system detected most clinically important (CIN2+) lesions and helped draw attention to suspicious areas, making it useful as a safety layer that can recover additional high-grade findings in difficult Normal/CIN1 and CIN1/CIN2 cases. For glandular lesions, the AI was less reliable and could both undercall and overcall these cases, but expert review corrected such high-consequence errors. Overall, our findings indicate that AI can support—but not replace—the pathologist in routine cervical biopsy diagnostics.

## 1. Introduction

Persistent infection with human papillomavirus (HPV) is the essential cause of almost all cervical cancers [1,2,3]. Cervical carcinogenesis progresses through precursor lesions within the transformation zone, most commonly classified as cervical intraepithelial neoplasia (CIN1–3) and, less frequently, glandular lesions such as adenocarcinoma in situ (ACIS) [4,5]. The introduction of HPV vaccination and the transition from cytology to HPV-based primary screening have substantially reduced cervical cancer incidence in many countries and altered the spectrum of cases referred to colposcopy—fewer classic high-grade lesions, more small or heterogeneous biopsies, and more low-grade/HPV-positive discordant findings [6,7,8,9]. Despite these advances, histopathologic evaluation of cervical biopsies remains the diagnostic gold standard for confirming high-grade disease and guiding treatment versus surveillance [5,10].

However, grading cervical biopsies is challenging in routine practice. Reactive changes, atrophy, metaplasia, tangential sectioning, and small biopsy size may obscure or mimic neoplasia. Inter- and intraobserver variability persists and is non-trivial: in ALTS, interobserver agreement was only moderate (κ ~0.46 for punch biopsies; κ ~0.49 for loop electrosurgical excision procedure [LEEP]; κ ~0.46 for monolayer cytology), with the poorest reproducibility in less severe categories [11]. This variability is most pronounced at the clinically consequential CIN1/CIN2 and CIN2/CIN3 thresholds and underpins current QA recommendations and standardized terminology [10,12]. In real-world laboratories, operational factors—high and fluctuating workload, variable training and experience among pathologists, and an evolving case mix in vaccinated populations—further contribute to inconsistent recognition of CIN2+ and to occasional under-calling of small high-grade foci [11,13,14,15]. Immunohistochemical markers (e.g., p16) can increase diagnostic confidence in equivocal lesions but add cost and turnaround time: at our laboratory, p16 requires re-cutting and an overnight immunostainer run, typically delaying sign-out by ~1–2 days, with reagent and technologist costs and limited instrument capacity that must be shared with other stains. Expert interpretation remains required [5,16]. As screening programs mature and vaccinated cohorts dominate, classic high-grade morphology becomes less frequent while borderline lesions become relatively more common, increasing pressure on cervical biopsy workflows and creating a rationale for digital pathology and AI-based attention guidance as quality-assurance tools [6,17,18].

Digital pathology now enables the use of whole-slide imaging (WSI) for diagnostic review and computational analysis [19,20]. Simultaneously, deep learning methods allow convolutional neural networks to learn morphological patterns directly from hematoxylin–eosin (H&E) images without manual feature engineering [21,22,23,24]. These models can detect candidate foci, aggregate tile-level predictions across gigapixel WSIs, and output slide-level grades aligned with diagnostic categories such as normal, CIN1, CIN2, CIN3, or invasive carcinoma [25,26]. In other organ systems, AI systems have demonstrated near-pathologist performance, particularly for quality assurance, case prioritization, and workload reduction [27,28,29,30].

Cervical histopathology is a particularly promising target for AI for three reasons. First, disease is localized to small epithelial compartments that are amenable to tile-based and multi-instance learning approaches [31]. Second, the clinical consequences of missed CIN2+ outweigh those of overtreatment, favoring high-sensitivity “safety-net” tools that can alert pathologists to suspicious areas [5,32]. Third, cervical tissue exhibits recurring architecture across laboratories, allowing cross-site generalization with appropriate curation and domain adaptation [33]. Nevertheless, existing studies differ widely in reference standards, case spectrum, and endpoints [31,34,35]. Most algorithms have been trained and tested mainly on squamous intraepithelial lesions, and performance is typically lower for glandular precursors such as ACIS, where morphology is subtler, lesion extent is smaller, and well-annotated training material is scarce [35,36,37,38]. This creates an unmet need to test AI on the full spectrum of cervical precursors, not only CIN2/3. Furthermore, slide-level probabilities alone are difficult to act upon; pathologists require interpretable visual outputs such as heatmaps or tile galleries embedded in familiar viewing environments [23,25]. Evaluation must therefore reflect real-world diagnostic decisions, particularly CIN2+ accuracy, impact on observer variability, and incremental benefit over unaided review [25,30,39].

Within this context, we developed an EagleEye-based workflow for diagnostic review of cervical biopsies using H&E WSIs. EagleEye is an AI-based, tile-level classification system that identifies epithelial regions, assigns per-tile predictions from normal epithelium to invasive carcinoma (including ACIS), and presents ranked tile galleries and WSI overlays to guide review. The intended role of EagleEye is decision support—improving sensitivity and consistency while retaining pathologist authority over p16 ordering, final diagnosis, and treatment recommendations.

This study evaluates EagleEye in a curated series of biopsies representing normal cervix, CIN1–3, and ACIS. We assessed (i) agreement between EagleEye and expert pathologists at the CIN2+ threshold using Cohen’s κ; (ii) sensitivity and specificity under alternative reference standards; (iii) variation in human performance across microscope and digital modalities; and (iv) spatial correspondence between EagleEye-highlighted regions and p16-positive epithelium. We hypothesized that EagleEye assistance would increase CIN2+ detection by resolving ambiguities at the CIN1/CIN2 boundary and surfacing small high-grade foci; that EagleEye alone would be less reliable for ACIS and would occasionally overcall squamous lesions as microinvasive carcinoma; and that pathologist verification of EagleEye-flagged regions (EE + P2) would be necessary to correct these high-consequence errors and restore accuracy for complex glandular lesions. In the following sections, we describe the study design and AI system (Section 2), present the diagnostic accuracy and agreement results (Section 3), and discuss implications, limitations, and future directions (Section 4 and Section 5).

## 2. Materials and Methods

### 2.1. Study Design and Case Selection

We performed a retrospective diagnostic accuracy study using archived cervical punch biopsies from the Department of Clinical Pathology, University Hospital of North Norway (UNN), Tromsø, Norway, serving the population-based cervical cancer screening program in Troms and Finnmark County, Norway. The study was conducted as part of a master’s thesis project and was therefore constrained by a single-centre design, a limited study period, and finite time and resources. A convenience sample of 99 cases was assembled to cover clinically relevant categories (19 normal, 20 CIN1, 20 CIN2, 20 CIN3, 20 adenocarcinoma in situ [ACIS]). In contemporary HPV-based cervical cancer screening, most punch biopsies are normal or CIN1, with relatively few CIN2/CIN3, very rare ACIS, and very few invasive carcinomas; a purely consecutive series would therefore have yielded too few high-grade and glandular lesions to meaningfully evaluate performance across the full histologic spectrum. For this reason, the cohort was deliberately spectrum-balanced to include all relevant diagnostic categories in approximately equal numbers.

Cases were identified in the laboratory information system SymPathy (Lifecare SymPathy; Tietoevry Care, Espoo, Finland) by diagnosis-based queries and then selected consecutively from archive hit lists until category quotas were met. To avoid incorporation bias, only biopsies that had not previously been diagnosed by P2 and had not been analysed by EagleEye were eligible. Normal, CIN1, and CIN2 cases were from 2023; most CIN3 cases were from 2023 with a small remainder from 2022 (to reach n = 20 under the eligibility criteria); ACIS cases, being rarer, were from 2019–2023. Diagnoses were made in routine practice according to the WHO Classification of Female Genital Tumours, 5th edition [40]. The original reporting pathologist is referred to as Pathologist 1 (P1). Because a large proportion of cervical biopsies at our department are routinely reported by senior gynecologic pathologists (P2-level readers), we specifically queried the archives to identify eligible cases reported by other pathologists (P1-level readers) in each diagnostic category to enable the planned comparisons between P1, P2, and EagleEye. No sampled cases were excluded. We acknowledge that this spectrum-balanced, convenience sampling and the single-centre setting may limit strict representativeness and external generalizability, but it was chosen to enable precise, per-category performance estimates within the available resources and to stress-test the system across the range from normal epithelium to high-grade squamous and glandular lesions (CIN3 and ACIS).

### 2.2. Slide Preparation and Digitization

Formalin-fixed, paraffin-embedded cervical biopsy blocks were sectioned and stained with hematoxylin and eosin (H&E) according to routine protocols at the Department of Clinical Pathology, University Hospital of North Norway (UNN). Whole-slide images (WSIs) were acquired on a Pannoramic-series scanner (3DHISTECH Ltd., Budapest, Hungary) at 40× equivalent magnification with a sampling rate of 0.25 µm/pixel. Files were stored on secure institutional servers in their native resolution and were accessed by the AI system and readers without downsampling, compression, or other pixel-level alteration.

### 2.3. AI System

EagleEye is a supervised, tile-based deep learning system for H&E whole-slide images (WSIs) intended for human-in-the-loop diagnosis. In this manuscript, we refer to it as “EagleEye” or “the algorithm”. The evaluated build (October 2021) used a ResNet-family convolutional neural network (CNN) for tile classification and was trained on archival cervical biopsies primarily from the University Hospital of North Norway (UNN), supplemented with cases from collaborating Norwegian sites with comparable H&E protocols and 3DHISTECH scanning [41]. Gynecologic pathologists (including P2) provided the reference labels; when available, prior p16 immunohistochemistry was used to refine the target class, covering the full spectrum from normal/reactive epithelium through CIN1–3 to ACIS and squamous cell carcinoma. A substantial subset of the training labels originated from cases originally diagnosed by P2 during routine work, and P2 contributed to tile-level annotations in the early development phase. However, none of the 99 study slides (or their corresponding tiles) were used for training, tuning, or calibration of EagleEye, and P2 did not annotate any of these study slides. The October 2021 codebase together with the pre-specified operating point was frozen before any human readings in this study.

At inference, EagleEye (i) detects and segments epithelial regions on the WSI; (ii) samples approximately 400–600 tiles at native resolution (0.25 µm/pixel); (iii) assigns each tile to one of the target classes (normal/reactive, metaplasia, CIN1, CIN2, CIN3, ACIS, squamous cell carcinoma, artifact/low quality); and (iv) aggregates tile-level outputs into a slide-level decision using a sensitivity-oriented operating point. A slide is considered algorithm-positive for CIN2+ when ≥15 tiles are classified as high-grade squamous/invasive carcinoma, or when a smaller but spatially coherent high-grade cluster is present and subsequently confirmed by expert review. For ACIS, ≥15 tiles classified as glandular high-grade are required. This ≥15-tile threshold was chosen heuristically from multi-year internal experience with EagleEye: with per-tile accuracy around 93%, requiring at least 15 concordant tiles reduces the impact of isolated false-positive tiles while maintaining sensitivity for clinically relevant foci. The threshold was not derived from formal ROC or decision-curve calibration and is therefore reported as a methodological limitation. Typical inference time on the institutional CPU server was approximately 5 min per biopsy.

**Figure 1:**
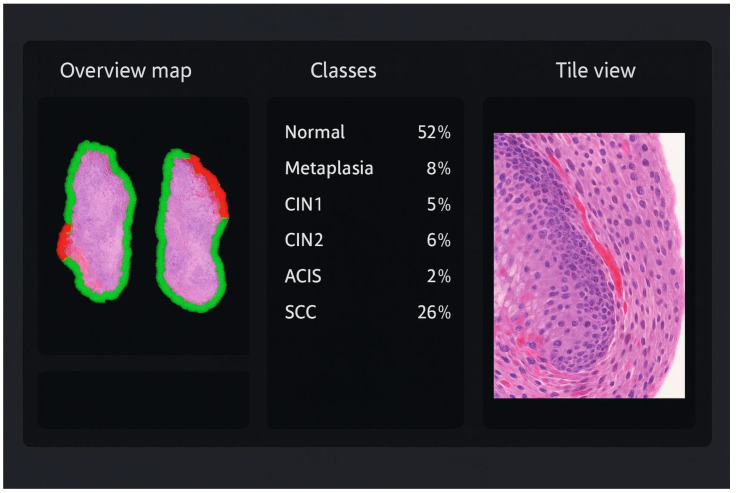
Illustration of the EagleEye user interface for a cervical biopsy. **Left**: overview map of the whole-slide image with colour-coded border and heatmap overlay; green indicates non-dysplastic epithelium, whereas yellow–orange–red highlights increasingly suspicious areas. **Centre**: class-distribution panel listing all diagnostic categories (normal/reactive, metaplasia, CIN1, CIN2, CIN3, ACIS, squamous cell carcinoma) with their colour codes and the proportion of tiles assigned to each class. **Right**: high-magnification tile view of the currently selected region, allowing the pathologist to scroll through AI-ranked hot spots and verify or refute the suggested classification.

The pathologist interacts with EagleEye through a split-screen graphical interface (Figure 1). On the left, an overview map of the WSI shows the biopsy fragments with a colour-coded border and overlaid heatmap, where green indicates non-dysplastic epithelium and progressively warmer colours (yellow–orange–red) indicate increasing suspicion of dysplasia or carcinoma. In the centre panel, all diagnostic categories (e.g., normal squamous epithelium, metaplasia, CIN1, CIN2, CIN3, ACIS, squamous cell carcinoma) are listed with their colour codes, together with the proportion of tiles assigned to each class by EagleEye and the number of tiles selected by the pathologist for documentation. On the right, the currently selected tile is shown at high magnification in the WSI viewer; by clicking on a diagnostic class (for example “SCC”) or using keyboard shortcuts, the pathologist can step through the tiles that EagleEye has ranked as most compatible with that diagnosis. Thus, the viewer exposes three interpretable outputs to the pathologist: (i) a ranked tile gallery with the most suspicious tiles first; (ii) a heatmap overlay highlighting algorithm-positive epithelium; and (iii) a class-distribution panel summarizing tile predictions. In the AI-assisted condition (EE + P2), the slide was first processed by EagleEye, and the pathologist then reviewed the WSI together with these outputs and issued the final diagnosis. EagleEye outputs functioned solely as decision support to direct attention towards candidate regions; the pathologist visually verified all AI flags and set the final diagnosis.

### 2.4. Human Readings

Two independent gynecologic pathologist roles participated. In this study, “P1” denotes the routine gynecologic pathologist role (i.e., the original reporting pathologist in routine practice; not a single named individual) who typically evaluates ~500 cervical biopsies per year, and “P2” denotes a senior gynecologic pathologist who evaluates ~2000 cervical biopsies per year. P2 has contributed to the development of EagleEye and to tile-level training labels in earlier phases of the project, but did not annotate or review any of the 99 study slides prior to this experiment. At our department, all high-grade diagnoses (CIN2+) initially signed by a P1-level reader undergo mandatory secondary review by another gynecologic pathologist before sign-out. Five diagnostic conditions were evaluated: (i) P1 (routine sign-out), (ii) EagleEye alone (EE), (iii) P2 by light microscope, (iv) P2 on digital WSI, and (v) P2 with AI assistance (EE + P2). “Pathologist” refers to P1/P2; “AI system” refers to EagleEye; and “EE + P2” denotes the human-in-the-loop condition.

P2 reviewed all cases under three conditions—microscope, digital WSI, and digital with AI assistance—in separate sessions with a ≥4-week washout and independently randomized case order for each session to minimize recall. P2 had no access to prior reports, clinical information, cytology, HPV results, earlier biopsies, deeper levels, or p16; only the index H&E slide was reviewed, and P2 was not the original reporting pathologist for any of the study cases. During the P2-microscope and P2-digital conditions, P2 was blinded to P1’s original diagnoses, to the artificial case balancing, to all EagleEye outputs, and could not access their own prior assessments. In the AI-assisted condition (EE + P2), P2 was given full access to EagleEye outputs (heatmaps, tile gallery, and class-distribution panel) but remained blinded to P1.

### 2.5. Reference Standards and Endpoints

We did not establish a single post hoc gold standard. All cases retained their original routine diagnosis signed out by P1; no diagnoses were altered for this study. Agreement and accuracy are therefore reported across the evaluated conditions (P1, EE, P2-microscope, P2-digital, EE + P2). P1 represents the routine sign-out that guided actual patient care. Patients were managed according to national guidelines outside this study; retrospective ancillary testing (e.g., p16 on discrepant cases) was not performed because the material was anonymized and the protocol did not allow patient-impacting interventions.

The primary endpoint was CIN2+ (CIN2, CIN3, ACIS, or invasive carcinoma) versus <CIN2 (normal/CIN1). Throughout the manuscript, “CIN2+” is used consistently to denote this composite endpoint, whereas “CIN2” refers only to the specific histologic diagnosis CIN2. Two reference standards were applied to mirror routine quality assurance and to quantify the incremental value of AI-assisted review:

(i) P1 (original sign-out) was used as reference to evaluate EagleEye and P2, reflecting retrospective QA against the diagnosis that guided management.
(ii) The AI-assisted consensus (EE + P2) was used as a comparative reference to evaluate P1, not as an independent gold standard but as a methodological construct to measure additional CIN2+ cases surfaced when an experienced gynecologic pathologist reviewed the WSI with AI guidance. Because EE + P2 incorporates the intervention under study, this comparison is subject to verification/incorporation bias and is presented strictly as an internal case-finding exploration rather than as a formal estimate of P1 diagnostic accuracy; all sensitivity/specificity estimates versus EE + P2 should therefore be interpreted as study-internal performance metrics, not as externally generalizable truth standards.

### 2.6. P16 Concordance Analysis

In 30 biopsies with prior p16 immunohistochemistry, concordance was assessed by side-by-side review of (i) the p16-stained glass slide at the microscope, (ii) the corresponding H&E WSI, and (iii) the EagleEye heatmap/tile view. The evaluator first identified p16-positive epithelial domains on the glass slide, then located the corresponding areas on the H&E WSI, and finally judged whether the AI highlights covered the same epithelial extent. Spatial overlap was recorded in predefined ordinal categories {0, 70, 80, 90, 100%}, representing the estimated proportion of the p16-positive epithelium marked by contiguous AI-positive tiles. The same AI operating point as in the main analysis (≥15 tiles for CIN2+ and ACIS) was used. No image registration, pixel-level quantification, or interobserver assessment was performed; this concordance assessment should therefore be interpreted as a single-evaluator, preliminary indication of biological plausibility rather than a formal validation.

### 2.7. Statistical Analysis

As defined above, the primary endpoint was CIN2+ (CIN2, CIN3, ACIS, or invasive carcinoma) versus <CIN2 (normal/CIN1). Agreement between readers and/or AI was quantified using Cohen’s κ with asymptotic 95% confidence intervals, supplemented by percent agreement to aid interpretation in this spectrum-enriched cohort. Cohen’s κ was used to assess interobserver reliability (how consistently different readers/conditions classified the same biopsies), while McNemar’s test was applied to paired 2 × 2 tables to evaluate directional marginal asymmetry (i.e., whether one reader/condition systematically upgraded or downgraded more cases than the comparator). Diagnostic accuracy at the CIN2+ threshold was expressed as sensitivity and specificity with exact (Clopper–Pearson) 95% confidence intervals, with explicit numerators and denominators provided in the Results and tables. Reclassification patterns between conditions (e.g., P1 vs. EE + P2) were summarized using cross-tabulations to show where CIN grade assignments changed (normal/CIN1/CIN2/CIN3/ACIS/SCC). The exploratory p16–AI concordance analysis was summarized as the proportion of cases with ≥70% visual overlap between p16-positive epithelium and AI-highlighted regions (exact 95% CI). All tests were two-sided with α = 0.05; no adjustment was made for multiple comparisons. Analyses were conducted in R (v4.5.1) and IBM SPSS Statistics for Windows, Version 29.0.1.0 (171) (Armonk, NY, USA: IBM Corp.); code for the R analyses is available upon request.

### 2.8. Efficiency and Human-Factor Endpoints

Reading time, time-to-sign-out, and perceived workload/stress were not recorded and were not predefined outcomes in this study; consequently, we cannot quantify any effect of EagleEye on diagnostic efficiency or workload, and any workflow-related implications discussed in the manuscript should be regarded as hypothesis-generating.

### 2.9. Sample Size and Precision

A total of 99 cases were selected to balance diagnostic categories and keep the blinded, multi-condition readings feasible. With the observed CIN2+ prevalence (~60–70%), this sample yields approximately moderate precision for binary accuracy estimates: for a sensitivity around 0.85 based on ~60 CIN2+ cases, the 95% CI is roughly ±9 percentage points; for a specificity around 0.90 based on ~35–40 non-CIN2+ cases, the 95% CI is roughly ±10 percentage points (Wilson method). Per-category subsets (n ≈ 20 per grade) have wider uncertainty (95% CI half-width ≈17–20 percentage points), limiting power for subgroup comparisons. Accordingly, the study was designed to provide feasible, spectrum-balanced point estimates at the CIN2+ threshold rather than to detect small between-modality differences.

### 2.10. Ethics and Data Governance

All material consisted of anonymized archived diagnostic samples. No findings affected patient care. The Regional Committee for Medical and Health Research Ethics (REK North) evaluated the project (REK 2018/485, 641038, 876436) and determined that it was exempt from the Health Research Act (§2), as it constituted method development and quality assurance. Institutional approval was granted.

## 3. Results

### 3.1. Study Cohort and Reader Classifications

**Figure 2:**
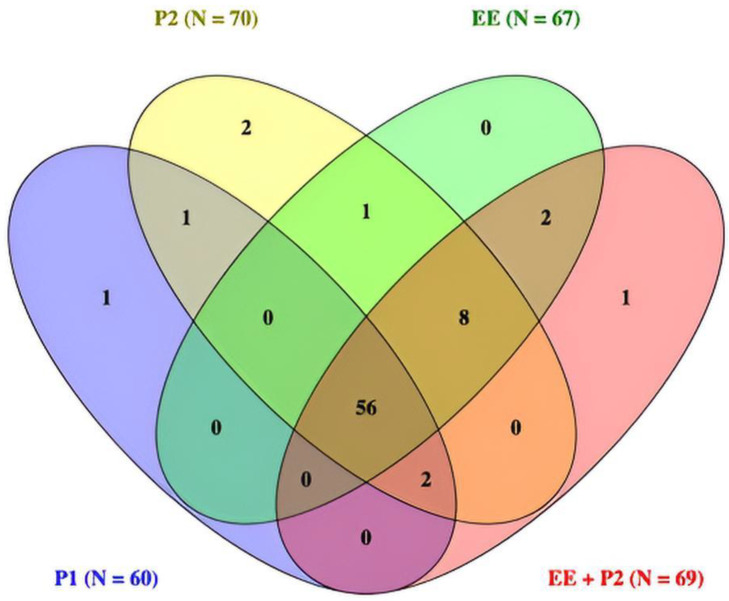
Venn diagram of CIN2+ calls across readers (n = 99). All four readers agreed on 56 cases. Eight additional cases were called CIN2+ by EE, P2, and EE + P2 but not by P1. Single-reader exclusives were rare (≤2 per set). Totals per reader: P1 = 60, P2 = 70, EE = 67, EE + P2 = 69.

A total of 99 cervical biopsies were evaluated across five diagnostic conditions: original sign-out (P1), second pathologist by microscope (P2-mic), second pathologist on digital WSI (P2-dig), EagleEye alone (EE), and AI-assisted second pathologist (EE + P2). The number of cases classified as CIN2+ was P1 = 60, P2 = 70, EE = 67, and EE + P2 = 69. Pairwise agreement between readers is summarized in Table 1, and diagnostic accuracy against alternative reference standards in Table 2 and Table 3. A Venn diagram (Figure 2) shows complete concordance across all four readers for 56 cases and eight additional CIN2+ cases jointly identified by EE, P2, and EE + P2 but not by P1; single-reader exclusives were rare (≤2). Detailed reclassification by original P1 category is provided in Table 4, Table 5, Table 6, Table 7 and Table 8. 

Consistent with its intended use as a high-sensitivity safety layer, EagleEye generated more false-positive CIN2+ flags than a human reader, but these served to focus the review; clinically important areas were unlikely to be overlooked under human-in-the-loop verification, and the resulting trade-off between sensitivity and specificity is quantified below.

### 3.2. Diagnostic Agreement at CIN2+ Threshold

We report agreement versus two pre-specified reference comparators—P1 (simulating routine sign-out) and, secondarily, EE + P2 (augmented review)—using Cohen’s κ with 95% confidence intervals and percent agreement as measures of interobserver reliability, and McNemar’s test to assess directional marginal asymmetry (i.e., whether one condition systematically upgraded or downgraded more cases than its comparator). Pairwise agreement at the CIN2+ threshold was moderate to almost perfect (Table 1).

**Table 1:** Pairwise diagnostic agreement at the CIN2+ threshold: percent agreement, Cohen’s κ (95% CI), and McNemar’s test.

Pairwise Comparison (Condition → Condition)	Percent Agreement (%)	κ (95% CI)	McNemar’s *p*-Value
P1 vs. EE (EE alone)	84.5	0.67 (0.52–0.82)	0.12
P2 microscope vs. P2 digital	93.8	0.86 (0.75–0.97)	0.68
P1 vs. P2 digital	89.7	0.78 (0.65–0.91)	0.03
EE (alone) vs. P2 digital	88.7	0.74 (0.60–0.89)	1.00
EE + P2 vs. P2 digital	90.7	0.79 (0.66–0.92)	1.00

P1 = routine gynecologic pathologist (~500 cervical biopsies/year); P2 = senior gynecologic pathologist (~2000/year). All P1 CIN2+ diagnoses were second-read by another gynecologic pathologist. P2 readings (microscope and digital) were separated by a ≥4-week washout interval, and case order was independently randomized for each session. McNemar’s test (with continuity correction) was used to assess directional disagreement at the CIN2+ threshold. N = 99 paired readings.

These results show strong within-reader consistency for P2 across modalities (percent agreement 93.8%, κ = 0.86) and good agreement between P1 and P2-digital (percent agreement 89.7%, κ = 0.78), indicating substantial interobserver reliability under both analogue and digital conditions. Relative to P2-digital, agreement with the algorithm increased from 88.7% (κ = 0.74) for EE alone to 90.7% (κ = 0.79) for EE + P2, indicating incremental value of human adjudication in a human-in-the-loop workflow, where the expert accepts or dismisses AI-suggested foci. By Landis–Koch benchmarks, P1 vs. EE (84.5% agreement, κ = 0.67) borders on “substantial,” and the remaining pairs are “substantial” to “almost perfect.”

Because κ is influenced by prevalence and marginal distributions, the high CIN2+ prevalence in this curated cohort (≈60–70%) likely attenuates κ despite high raw agreement; percent agreement is therefore reported alongside κ to aid interpretation. McNemar’s test showed a significant directional shift only for P1 vs. P2-digital (*p* = 0.027), demonstrating that the experienced digital review more often upgraded cases to CIN2+ than P1, consistent with the study’s case-finding rationale. All other pairs (P1 vs. EE, P2 microscope vs. P2 digital, EE vs. P2 digital, and EE + P2 vs. P2 digital) had few and balanced disagreements (*p* ≥ 0.12), indicating no systematic bias toward upgrading or downgrading. For transparency, we therefore summarize both agreement metrics (percent agreement, κ) and accuracy metrics (sensitivity/specificity) versus both P1 and, secondarily, EE + P2; neither comparator constitutes an independent gold standard.

### 3.3. Diagnostic Accuracy Analyses

#### 3.3.1. AI Compared with Original Diagnosis (Reference = P1)

Using P1 as reference, EE detected 56/60 CIN2+ cases with a specificity trade-off (Table 2).

**Table 2:** Diagnostic performance of EE vs. P1 (Reference = P1).

	EE: Normal/CIN1	EE: CIN2+	Sum
P1: Normal/CIN1	28	11	39
P1: CIN2+	4	56	60
Sum	32	67	99

Sensitivity = 93.3% (56/60; 95% CI 83.8–98.2); specificity = 71.8% (28/39; 95% CI 55.1–85.0).

Against P1 as reference, EE identified 56/60 CIN2+ cases (sensitivity 93.3%) with specificity 71.8% (28/39), corresponding to 11 false positives (P1 <CIN2) and 4 false negatives. Overall accuracy was 84.8% (84/99); PPV 83.6% (56/67); NPV 87.5% (28/32). This illustrates the intended operating characteristics of EagleEye: a high-sensitivity safety layer that preferentially upgrades borderline low-grade cases (Normal/CIN1) at the CIN1/CIN2 threshold, rather than missing treatment-relevant disease.

#### 3.3.2. Original Diagnosis Compared with AI-Assisted Review (Reference = EE + P2)

Using EE + P2 as a comparative internal reference, we examined how many CIN2+ cases surfaced only after AI-assisted review (Table 3).

**Table 3:** Diagnostic performance of P1 vs. EE + P2 (Reference = EE + P2).

	P1: Normal/CIN1	P1: CIN2+	Sum
EE + P2: Normal/CIN1	31	0	31
EE + P2: CIN2+	11	57	68
Sum	42	57	99

Sensitivity = 83.8% (57/68; 95% CI 72.9–91.6); specificity = 100% (31/31; 95% CI 88.8–100).

Relative to EE + P2, P1 classified 57/68 CIN2+ cases as high grade (apparent sensitivity 83.8%) and did not upstage any EE + P2 <CIN2 cases to CIN2+ (apparent specificity 100%). These estimates are subject to incorporation/verification bias because EE + P2 includes the AI intervention under study and should therefore be interpreted as study-internal case-finding metrics rather than as externally valid measures of P1 diagnostic accuracy. In line with the Venn diagram, the 11 discrepant CIN2+ cases are concentrated near the CIN1/CIN2 threshold and illustrate the types of lesions that may be “recovered” when an experienced gynecologic pathologist reviews the WSI with access to EagleEye outputs, while maintaining a very low false-positive rate at the treatment threshold.

#### 3.3.3. Expert Digital Review Compared with AI-Assisted Review (P2-Digital vs. EE + P2)

Using EE + P2 as a comparative internal reference, we also examined whether AI assistance changed the marginal CIN2+ rate for the experienced gynecologic pathologist (P2). The 2 × 2 cross-tabulation between P2-digital and EE + P2 at the CIN2+ threshold is shown in Table 4.

**Table 4:** Diagnostic cross-tabulation of P2-digital vs. EE + P2 at the CIN2+ threshold (Reference = EE + P2).

	P2-Digital: Normal/CIN1	P2-Digital: CIN2+	Sum
EE + P2: Normal/CIN1	26	4	30
EE + P2: CIN2+	5	64	69
Sum	31	68	99

Sensitivity = 92.8% (64/69; 95% CI 86.6–98.9); specificity = 86.7% (26/30; 95% CI 74.5–98.8).

With EE + P2 as internal reference, P2-digital classified 64/69 CIN2+ cases as high grade (apparent sensitivity 92.8%) and 26/30 <CIN2 cases as low grade (apparent specificity 86.7%). Discordant classifications were nearly symmetric (4 apparent false positives and 5 apparent false negatives for P2-digital), and McNemar’s test showed no evidence of a directional shift (*p* = 1.00). These findings indicate that, for the high-volume expert reader, the AI-assisted workflow (EE + P2) did not significantly alter the overall CIN2+ calling rate relative to unaided digital review, but rather redistributed a small number of borderline cases in both directions. In line with our overall framing, these comparisons are interpreted as internal case-finding characteristics of the human-in-the-loop workflow rather than as formal stand-alone accuracy estimates for either condition.

### 3.4. Reclassification by Assessment Condition (Pathologist and Algorithm)

Reclassification patterns across readers (pathologists) are shown in Table 4, Table 5, Table 6, Table 7 and Table 8.

**Table 5:** Reclassification of cases originally signed out as Normal by P1 (n = 19).

Call	P2 (Microscope)	P2 (Digital)	EE Alone	EE + P2
Normal	5	6	2	2
CIN1	13	11	13	14
CIN2	1	2	4	3
CIN3	0	0	0	0
ACIS	0	0	0	0
SCC	0	0	0	0

EE = EagleEye algorithm (not a human reader); EE + P2 = P2 reviewing WSIs with access to EE outputs.

P1-Normal (n = 19). Reclassifications clustered at the low end of the spectrum (Table 5). Across all alternative readings, most P1-Normal biopsies were shifted to CIN1, and a small subset was upgraded to CIN2. P2 (microscope/digital) called 5–6 cases Normal, 11–13 CIN1, and 1–2 CIN2, indicating that additional epithelial atypia was recognizable on re-review and that modality had minimal effect. EE similarly favored CIN1 (n = 13) but flagged 4 cases as CIN2; 3 of these upgrades were retained after adjudication in the EE + P2 condition. Thus, even among P1-Normal cases, a minority harbored foci that a second gynecologic pathologist and the AI-assisted workflow interpreted as meeting CIN2 criteria.

**Table 6:** Reader reclassification of cases originally signed out as CIN1 by P1 (n = 20).

Call	P2 (Microscope)	P2 (Digital)	EE Alone	EE + P2
Normal	1	1	0	0
CIN1	11	10	13	12
CIN2	8	9	7	8
CIN3	0	0	0	0
ACIS	0	0	0	0
SCC	0	0	0	0

P1-CIN1 (n = 20). Reclassifications were almost entirely confined to the CIN1↔CIN2 boundary with no shifts to CIN3, ACIS, or carcinoma (Table 6). P2 upgraded 8–9/20 cases to CIN2 (microscope 8; digital 9) and downgraded 1 case to Normal, again showing minimal modality effect. EE classified 13 as CIN1 and 7 as CIN2; after adjudication, EE + P2 yielded 12 CIN1 and 8 CIN2. These patterns confirm that the CIN1/CIN2 threshold is the most unstable category in this dataset and that most of the 11 CIN2+ cases “recovered” by the AI-assisted workflow (cf. Table 3) originate from these borderline P1-Normal and P1-CIN1 groups.

**Table 7:** Reader reclassification of cases originally signed out as CIN2 by P1 (n = 20).

Call	P2 (Microscope)	P2 (Digital)	EE Alone	EE + P2
Normal	0	0	0	0
CIN1	1	0	1	1
CIN2	17	18	18	18
CIN3	2	2	0	0
ACIS	0	0	1	1
SCC	0	0	0	0

P1-CIN2 (n = 20). Classifications were highly stable across readers: 17–18/20 remained CIN2 in all conditions, with no downgrades to Normal and only rare shifts to CIN1 (0–1) (Table 7). P2 upgraded 2 cases to CIN3 under both modalities, indicating minor upward drift at the high-grade boundary. EE kept 18 as CIN2 and upgraded 1 to ACIS, which was confirmed after adjudication (EE + P2). Overall, the CIN2 category showed strong stability with limited bidirectional reclassification and concordant identification of a single glandular lesion.

**Table 8:** Reader reclassification of cases originally signed out as CIN3 by P1 (n = 20).

Call	P2 (Microscope)	P2 (Digital)	EE Alone	EE + P2
Normal	0	0	0	0
CIN1	0	0	0	0
CIN2	2	1	5	5
CIN3	14	15	8	9
ACIS	0	0	0	0
SCC	4	4	7	6

P1-CIN3 (n = 20). P2 largely maintained the CIN3 diagnosis (14–15/20) with minimal modality effect and only rare downgrades to CIN2 (1–2) (Table 8). EE alone redistributed these cases toward more aggressive categories, assigning fewer CIN3 (8/20) and more SCC (7/20) or CIN2 (5/20). After expert adjudication, 6 of the 7 AI-flagged SCC cases were retained in EE + P2, indicating that most AI-triggered upgrades to invasion reflected true high-consequence findings. This pattern shows that the EagleEye model has a deliberate propensity to alert on potential invasion, and that human review can filter the few overcalls while preserving the safety-net function.

P1-ACIS (n = 20). Pathologists showed high concordance on glandular disease (P2: 19–20/20 ACIS, with only a single digital downgrade), whereas EE alone under-called ACIS (13/20) and redistributed the remainder in both clinically unfavorable directions—downward to CIN1 (n = 3), which represents a potential false negative at the treatment threshold, and upward to SCC (n = 4), which represents a false positive invasive call (Table 9). After expert adjudication in the EE + P2 condition, ACIS was restored to 18/20 and SCC was reduced to 1/20, demonstrating that the human reviewer corrected virtually all high-consequence AI errors. Taken together, these findings show that EE is less reliable for rare, complex glandular lesions than for squamous CIN, and that the intended human-in-the-loop workflow is essential to maintain accuracy when training data are limited.

**Table 9:** Reader reclassification of cases originally signed out as ACIS by P1 (n = 20).

Call	P2 (Microscope)	P2 (Digital)	EE Alone	EE + P2
Normal	0	1	0	0
CIN1	0	0	3	1
CIN2	0	0	0	0
CIN3	0	0	0	0
ACIS	20	19	13	18
SCC	0	0	4	1

### 3.5. Venn Diagram of CIN2+ Concordance

The overlap of CIN2+ classifications across P1, P2, EE, and EE + P2 is shown in Figure 2.

### 3.6. Exploratory Concordance with p16 Immunohistochemistry

Among 30 cases with available p16 staining, visual overlap ≥70% between p16-positive epithelium and AI highlights at the predefined operating point was observed in 22/30 cases (73.3%). This supports biological plausibility of the AI detections. Given the lack of image registration, single-evaluator scoring, and ordinal (semi-quantitative) bins, these findings are exploratory and were not used to alter classifications.

## 4. Discussion

### 4.1. Principal Findings

This study demonstrates incremental value of a human-in-the-loop AI workflow rather than AI superiority. Using P1 (original sign-out) as the primary reference, EagleEye functioned as a high-sensitivity safety layer (sensitivity 93.3%, specificity 71.8%), indicating that the algorithm can surface additional suspicious foci in this spectrum-enriched cohort. When we instead used the AI-assisted consensus (EE + P2) as an internal comparative construct, P1 showed 100% specificity but lower apparent sensitivity (83.8%), because 11 CIN2+ cases were classified as high grade only when an experienced gynecologic pathologist reviewed the WSI with AI guidance. These EE + P2-based estimates are subject to incorporation/verification bias and should be interpreted as study-internal case-finding signals rather than externally valid measures of P1 diagnostic accuracy. Taken together, the clinically relevant message is that AI assistance appears to standardize and elevate detection in the most unstable categories (P1-Normal and P1-CIN1), while preserving pathologist authority, rather than that AI outperforms an individual pathologist across the board.

### 4.2. Agreement, Reference Effects, and Relation to Prior Work

Pairwise agreement was high across all human conditions (percent agreement 89–94%; κ up to 0.86), indicating that WSI did not degrade human performance [42]. The only significant directional shift on McNemar’s test was for P1 vs. P2-digital, showing that an experienced, blinded digital review upgraded more cases to CIN2+ than the original sign-out—exactly the type of case-finding shift the AI-assisted workflow is designed to support. Agreement between EE and P2-digital improved further when AI outputs were adjudicated (EE + P2 κ = 0.79 vs. EE-alone κ = 0.74), indicating that the main effect of AI is to surface additional suspicious foci and let the expert accept or dismiss them [43,44]. Because the cohort was spectrum-enriched (≈60–70% CIN2+), κ is likely attenuated; percent agreement is therefore reported alongside κ to show that reliability remained in the “substantial–almost perfect” range. None of the comparators represent an independent gold standard; EE + P2 was used purely as an internal, methodological construct to quantify incremental case-finding over P1 under spectrum-enriched conditions, not as a definitive reference for diagnostic accuracy. Notably, our κ estimates exceed those reported in ALTS (≈0.46–0.49 across cytology, punch biopsy, and LEEP) [11], but direct comparison is limited by design differences (single-center histology study vs. multicenter ALTS, spectrum-balanced sampling with high CIN2+ prevalence, fewer readers, and distinct QA/blinding procedures). Taken together, these findings support higher internal consistency under contemporary workflows and standardized criteria, while acknowledging these contextual differences.

### 4.3. Diagnostic Reclassification Patterns and Clinical Implications

The reclassification tables show where the incremental value of the AI-assisted workflow arises within this spectrum-enriched cohort. Most of the 11 CIN2+ cases that were classified as CIN2+ by EE + P2 but not by P1 came from P1-Normal and P1-CIN1 biopsies that contained small, equivocal, or heterogeneous epithelial atypia—precisely where interobserver variability is highest. In P1-CIN3 cases, AI deliberately “over-alerted” by flagging possible invasion; expert review retained 6/7 of these SCC calls, supporting AI as a critical alert function for high-consequence disease. In ACIS, the EagleEye algorithm alone was less reliable, but the human-in-the-loop workflow restored ACIS to 18/20 and eliminated most false invasive calls, confirming that pathologist verification is indispensable for rare glandular lesions where training data are sparse. Overall, these internal reclassification patterns support a workflow in which AI raises the floor of detection and the pathologist protects specificity [45], while recognizing that the magnitude of case-finding gain must be validated in unselected, real-world biopsy cohorts.

### 4.4. Performance in Glandular Disease

Performance on glandular lesions was clearly weaker than on squamous disease. Pathologists showed near-complete concordance for ACIS (19–20/20), while EE alone under-called ACIS (13/20) and redistributed the remainder in both clinically unfavorable directions (to CIN1 and to SCC). After human adjudication in the EE + P2 condition, ACIS was restored to 18/20 and false invasive calls were largely removed (SCC 1/20). This pattern is important: it shows that the model can surface atypical epithelial compartments but cannot on its own discriminate reliably within the glandular spectrum, most likely because ACIS is rare, morphologically subtle, and underrepresented in the training set. The fact that accuracy was only recovered when the pathologist reviewed AI-highlighted regions is therefore not a weakness of the workflow but its main validation—complex, low-prevalence lesions still require expert judgment, and AI is best used to ensure they are not overlooked rather than to replace the pathologist. Future versions should include targeted ACIS enrichment and domain-adapted training, but the intended role remains decision support for rare glandular lesions [35,46].

### 4.5. Biological Plausibility and Workflow Integration

An exploratory comparison with p16 immunohistochemistry showed visual overlap between AI heatmaps/tiles and p16-positive domains in 73.3% of cases. Although qualitative and not a substitute for immunostaining, this concordance suggests that the EagleEye model highlights morphologic regions enriched for biologic transformation. Future work should quantify spatial correspondence with image registration (e.g., Jaccard/Dice) and evaluate whether AI-guided review can reduce discretionary p16 ordering without compromising accuracy [45]. From an implementation perspective, recent reviews emphasize that responsibility should remain with the pathologist and that AI is best deployed to streamline workflows, prioritize high-risk material, and reduce variance rather than replace expert judgment [47]. National efforts to standardize safe deployment—governance, calibration across scanners and stains, documentation, and monitoring—are timely and necessary [48]. As vaccinated cohorts reshape case mix and reduce the prevalence of classic high-grade morphology, transparent AI outputs (tile galleries/overlays) reviewed by experts may help maintain sensitivity and consistency, prioritize scarce expert attention, and support efficient diagnostic pathways [6,7,49].

### 4.6. Intended Use and Operating Point

EagleEye is designed for attention guidance rather than autonomous diagnosis. We selected an operating point favoring sensitivity to minimize the chance of missed CIN2+ lesions, accepting more false-positive highlights. In practice, false positives are filtered by the pathologist during rapid visual verification, while true-positive flags can improve case-finding (including rare invasive foci within CIN3). This framing aligns with decision-support deployment where safety (miss rate) is prioritized over stand-alone precision.

### 4.7. Implications and Future Work

Prospective implementation studies are needed to quantify the workflow impact of EagleEye under realistic routine conditions. In the present retrospective design, previously reported slides were re-read in multiple blinded sessions (microscope, digital WSI, and AI-assisted), which does not reflect a real-time diagnostic pathway and therefore did not allow meaningful measurement of reading time, workload, or human-factor end-points; any statements about efficiency and workload in this manuscript should thus be regarded as hypothesis-generating. At the time of the study, our department was not fully digitized and routine practice was still based on analogue slide reading in the microscope; EagleEye was operated as a stand-alone research tool rather than being integrated into the laboratory information system and primary WSI viewer. As we transition to a fully digital workflow in which all slides are scanned and EagleEye outputs (heatmaps, tile galleries, and provisional class distributions) can be made available at the time of primary review, future studies should instrument workflows to log per-case reading time and micro-interactions (e.g., time on flagged regions vs. unflagged scan), and collect standardized measures of perceived workload. Such data are needed to determine whether AI assistance genuinely reduces search burden or adds verification overhead, how it influences reader stress and diagnostic reliability, and whether concordance between a pathologist and EagleEye could safely replace routine second human review for selected high-grade cases.

Beyond diagnostic accuracy, our findings support EagleEye as a safety-net decision-support tool that can elevate CIN2+ detection in diagnostically challenging cases, while allowing the expert to filter AI overcalls and retain control over the final diagnosis. In practice, such a system could contribute to more consistent triage decisions, reduce the risk of missed high-grade lesions, and potentially optimize the targeted use of ancillary testing (e.g., p16) by directing attention to the most suspicious foci. However, several steps are required before broad clinical adoption: (i) prospective, external, multi-site validation on unselected screening and referral cohorts and across scanners/staining protocols to address domain shift and confirm robustness; (ii) rigorous quantification of workflow effects, including time-to-diagnosis, reading time, targeted p16 use on AI-flagged areas, and prioritization of high-risk cases; and (iii) further model development with enriched, domain-adapted training material for rare glandular lesions to improve autonomous suggestions while preserving transparent, interpretable outputs (tile galleries and WSI overlays). If these conditions are met, human-in-the-loop AI systems such as EagleEye may become an integral component of quality-assured cervical histopathology in modern screening programs.

### 4.8. Strengths

Strengths include population-based screening material from Troms and Finnmark; high-quality reference diagnoses (double reading for CIN2+, consensus for equivocal CIN1, and access to longitudinal history, deeper levels, and p16 when indicated); adjudication by an experienced external gynecologic pathologist (P2); standardized WSI acquisition and staining; multiple reader conditions (P1, P2-mic, P2-dig, EE, EE + P2); transparent operating characteristics (2 × 2 tables, κ with CIs); and an exploratory p16 comparison supporting biological plausibility within a human-in-the-loop design. Reader experience likely influences both agreement and accuracy; our setting included a high-volume senior gynecologic pathologist (P2) and routine-volume gynecologic pathologists (P1), with mandatory secondary review of all P1 CIN2+ calls. This may limit generalizability to settings without such workflows. The study was conducted as a single-centre master’s thesis project with constrained time and resources, which necessitated a spectrum-balanced convenience sample of 99 cases rather than an unselected screening cohort. Residual familiarity of individual cases cannot be entirely excluded, but blinding to prior investigations and a ≥4-week washout with re-randomized order make material recall unlikely across 99 cases.

The deliberately spectrum-balanced cohort, with enrichment for CIN2+ and ACIS, is also a strength in that it stress-tested the AI system across the full squamous–glandular spectrum, although the implications for generalizability are discussed under Limitations.

### 4.9. Limitations

First, this was an internal, single-center study from a single Norwegian university hospital, with its own screening routines, case mix, HPV vaccination coverage and referral patterns, using one H&E protocol and one scanner. The AI was trained and validated largely on local material annotated by the same gynecologic pathologist who also participated as reader. The relatively small sample size and the fact that the work was carried out within the scope of a master’s thesis further limit external generalizability and precluded inclusion of a broader, more heterogeneous case mix. External, multi-site validation with heterogeneous staining/scanning and larger, unselected screening and referral cohorts is therefore required before translation. Second, the dataset was intentionally spectrum-balanced with a much higher CIN2+/ACIS prevalence than in routine screening or ordinary referral cohorts; this spectrum bias inflates PPV/NPV and may also influence κ, so predictive values reported here should be considered study-internal and would be lower at 5–10% CIN2+ prevalence. Consequently, all sensitivity/specificity estimates and the observed “recovery” of 11 additional CIN2+ cases should be interpreted as internal performance under spectrum-enriched conditions rather than as a direct estimate of real-world clinical gain in routine biopsy cohorts. Third, the AI operating point (AI-positive defined as ≥15 concordant high-grade tiles, or a smaller coherent cluster on expert confirmation) was heuristically chosen from local experience rather than derived from formal ROC or decision-curve analysis. In the current tile-based implementation of EagleEye, there is no continuous slide-level probability score or tunable operating point that would allow post hoc ROC/decision-curve calibration without re-designing and re-training the model; future versions with probabilistic slide-level outputs should enable such formal threshold optimization. Fourth, p16–AI correspondence was assessed only qualitatively, without image registration, pixel-level metrics, or interobserver assessment, and therefore supports biological plausibility only. Additional limitations include information asymmetry favoring P1 (clinical context, deeper levels, p16) versus EE/P2 (single H&E level, blinded context); modest sample size limiting subgroup analyses, especially for ACIS; the absence of a unified histologic gold standard (P1 reflects routine sign-out, EE + P2 was an analytical construct to quantify incremental case-finding and is subject to incorporation bias); and the fact that we did not measure efficiency or human-factor endpoints (reading time, workload, alert fatigue), which limits the strength of any inferences about workflow utility and should be regarded as hypothesis-generating only. The evaluated EagleEye build was the October 2021 version; newer releases would require re-validation.

## 5. Conclusions

In this spectrum-enriched series of 99 digitized cervical biopsies, a human-in-the-loop AI workflow (EagleEye) achieved high case-finding for treatment-relevant disease at the CIN2+ threshold while pathologists retained full diagnostic authority. Compared with the original sign-out (P1), EagleEye reached 93.3% sensitivity (specificity 71.8%); using the AI-assisted read (EE + P2) as a methodological comparator, P1 showed 83.8% sensitivity and 100% specificity, indicating that AI guidance helped surface additional CIN2+ lesions mainly in borderline P1-Normal/CIN1 biopsies and subtle invasive candidates within P1-CIN3. Agreement across readers and conditions was substantial to almost perfect (percent agreement 89–94%; κ up to 0.86), and diagnostic performance was strongest for squamous CIN, with ACIS highlighting the intended dependence on expert adjudication in a human-in-the-loop design.

Taken together, these findings support EagleEye as a safety-net decision-support tool that can reduce the risk of missed high-grade lesions and promote more consistent grading of cervical biopsies, while final diagnostic responsibility remains with the pathologist. External multi-site validation and prospective workflow studies will be needed to confirm generalizability and quantify the impact of EagleEye on routine diagnostic pathways before broad clinical implementation.

## Data Availability

The original contributions presented in this study are included in the article/Supplementary Material. Further inquiries can be directed to the corresponding author. Whole-slide images (WSIs) and any associated clinical metadata cannot be shared publicly due to institutional policy and Norwegian privacy regulations governing diagnostic patient material. Analysis code (R scripts used for agreement and accuracy calculations) is available from the corresponding author upon reasonable request.

## Supplementary Materials

The following supporting information can be downloaded at: https://www.mdpi.com/article/10.3390/cancers17233808/s1, Supplementary File S1. Minimal dataset (Excel) underlying Table 4, Table 5, Table 6, Table 7, Table 8 and Table 9.

## Abbreviations

The following abbreviations are used in this manuscript:

Abbreviation	Definition
ACIS	Adenocarcinoma in situ
AI	Artificial intelligence
CI	Confidence interval
CIN	Cervical intraepithelial neoplasia
CIN1	CIN grade 1 (low-grade)
CIN2	CIN grade 2 (high-grade)
CIN2+	CIN2, CIN3, ACIS and cervical cancer
EE	EagleEye (AI system)
EE + P2	EagleEye with pathologist adjudication
H&E	Hematoxylin and eosin
HPV	Human papillomavirus
IHC	Immunohistochemistry
κ	Cohen’s kappa
LEEP/LLETZ	Loop electrosurgical excision procedure/Large loop excision of the transformation zone
NPV	Negative predictive value
P1	Routine reporting gynecologic pathologist (original sign-out)
P2	Senior gynecologic pathologist (independent second reader)
PPV	Positive predictive value
REK	Regional Committee for Medical and Health Research Ethics (Norway)
SCC	Squamous cell carcinoma
UNN	University Hospital of North Norway
WHO	World Health Organization
WSI	Whole-slide image/imaging
